# Advanced Functional Wound Dressings in Precision Surgery: Immunometabolic Reprogramming, Bioadaptive Biomaterials, and Intelligent Regenerative Interfaces

**DOI:** 10.3390/ijms27135772

**Published:** 2026-06-26

**Authors:** Tomasz Urbanowicz, Alessandro Mattina, Judyta Cielecka-Piontek, Giuseppe Maria Raffa, Calogera Pisano, Ewelina Grywalska, Anna Hymos, Mansur Rahnama, Mariusz Kowalewski, Piotr Suwalski, Marek Jemielity, Zbigniew Krasiński

**Affiliations:** 1 Cardiac Surgery and Transplantology Department, Poznan University of Medical Sciences, ½ Długa Street, 61-848 Poznan, Poland; 2 Department of Research, IRCCS-ISMETT (Mediterranean Institute for Transplantation and Specialized Therapies), 90127 Palermo, Italy; 3 Department of Pharmacognosy and Biomaterials, Poznan University of Medical Sciences, Rokietnicka 3, 60-806 Poznan, Poland; 4 Department of Precision Medicine in Medical Surgical and Critical Area (Me.Pre.C.C.), University of Palermo, 90134 Palermo, Italy; 5 Department of Experimental Immunology, Medical University of Lublin, 20-093 Lublin, Poland; 6 Department of Dental Surgery, Medical University of Lublin, 6 Chodźki Street, 20-093 Lublin, Poland; 7 Department of Cardiac Surgery, Center of Postgraduate Medical Education, Central Clinical Hospital of the Ministry of Interior, 02-507 Warsaw, Poland; 8 Department of Vascular, Endovascular Surgery, Angiology and Phlebology, Poznan University of Medical Science, ½ Dluga Street, 61-848 Poznan, Poland

**Keywords:** advanced wound dressings, immunometabolism, regenerative biomaterials, smart hydrogels, macrophage reprogramming, ferroptosis, extracellular vesicles, bioelectronic wound interfaces, precision regenerative medicine, wound healing

## Abstract

Postoperative wound complications remain a major cause of morbidity, prolonged hospitalization, increased healthcare costs, and reduced quality of life. While traditional wound dressings functioned primarily as passive barriers against contamination and exudate, advances in wound biology have transformed surgical wound management. Tissue repair is now recognized as a dynamic immunometabolic process involving coordinated interactions among immune cells, stromal populations, extracellular matrix remodeling, mechanotransduction, mitochondrial function, redox balance, microbial ecology, and bioelectrical signaling. Consequently, modern wound dressings are increasingly designed as bioactive systems capable of actively modulating the wound microenvironment. Recent developments in biomaterials science, immunoengineering, nanotechnology, extracellular vesicle biology, bioelectronics, and artificial intelligence have enabled the creation of advanced wound platforms, including stimuli-responsive hydrogels, immunomodulatory biomaterials, nanozyme-based dressings, conductive scaffolds, oxygen-generating matrices, extracellular vesicle-loaded systems, and biosensor-integrated interfaces. Therapeutic strategies are progressively shifting from antimicrobial-focused approaches toward immune-regenerative modulation targeting chronic inflammation, mitochondrial dysfunction, ferroptosis, cellular senescence, and impaired mechanobiological signaling. This review examines emerging surgical wound dressings from mechanistic, translational, and biomaterial perspectives, highlighting current innovations, translational challenges, and future directions. Collectively, these technologies may enable intelligent therapeutic systems capable of sensing and directing tissue regeneration in real time.

## 1. Introduction

Surgical wound healing represents one of the most intricate regenerative processes in human physiology. Successful restoration of tissue integrity requires tightly coordinated interactions among inflammatory mediators, innate and adaptive immune cells, fibroblasts, endothelial populations, extracellular matrix components, and metabolic signaling pathways [1,2]. Disruption of these finely regulated processes may result in delayed healing, chronic inflammation, excessive fibrosis, wound dehiscence, or surgical site infection (SSI), ultimately compromising postoperative recovery and long-term clinical outcomes [3,4].

Despite substantial progress in perioperative antimicrobial prophylaxis, minimally invasive surgery, and critical care medicine, wound-related complications remain a major global healthcare burden [5,6,7]. Surgical site infections alone account for a considerable proportion of postoperative morbidity, prolonged hospitalization, reoperation, and mortality [8,9,10]. Beyond systemic antimicrobial prophylaxis, local antimicrobial biomaterial strategies have emerged as valuable adjuncts for infection prevention. In a single-center clinical study, Kaczmarek et al. demonstrated that incorporating a gentamicin-collagen sponge into a multicomponent prevention strategy was safe, effective, and cost-effective for reducing infections in cardiac implantable electronic devices, supporting the clinical utility of localized antimicrobial delivery systems in high-risk surgical settings [11].

The burden is particularly pronounced in patients with diabetes mellitus, obesity, peripheral vascular disease, chronic kidney disease, malignancy, and immunosuppressive conditions [12]. Moreover, aging populations and increasing prevalence of metabolic disorders continue to exacerbate the incidence of impaired wound healing worldwide [13,14].

Conventional wound dressings, including gauze, hydrocolloids, and absorptive materials, were primarily developed to provide mechanical protection and moisture balance [15,16]. Although these materials provide basic wound coverage, they inadequately address the complex biological processes underlying postoperative tissue repair. Increasing evidence indicates that wound healing is fundamentally governed by dynamic immunological, metabolic, microbiological, and mechanobiological interactions rather than simple structural closure alone.

This evolving understanding has driven a major paradigm shift in wound dressing design. Modern wound dressings are increasingly conceptualized as biologically interactive therapeutic systems engineered to actively regulate inflammation, oxidative stress, angiogenesis, cellular metabolism, extracellular matrix remodeling, and microbial homeostasis [17,18]. The convergence of biomaterials science, nanotechnology, regenerative medicine, bioelectronics, and systems biology has therefore generated a new generation of “smart” wound platforms capable of interacting directly with wound physiology.

Importantly, recent advances have further revealed that pathological wound healing is strongly associated with dysregulated immunometabolism, mitochondrial dysfunction, ferroptotic cell death, senescence-associated secretory phenotypes, and maladaptive mechanotransduction [19,20,21]. Consequently, future wound dressings may derive their therapeutic efficacy not merely from antimicrobial activity or moisture retention but from precise modulation of the biological networks governing tissue regeneration.

Importantly, the emerging paradigm in regenerative wound management increasingly suggests that failure of tissue repair arises not merely from inadequate wound coverage or microbial contamination, but from insufficient control over the immunometabolic architecture governing tissue regeneration [22]. In this context, advanced wound dressings are progressively evolving from passive biomaterials toward biologically interactive regulatory systems capable of dynamically modulating inflammatory signaling, mitochondrial homeostasis, mechanotransduction, cellular metabolism, and intercellular communication. Consequently, the future of surgical wound management may depend less on structural wound protection and more on precise spatiotemporal regulation of the molecular networks that determine regenerative competence.

This review examines emerging advanced wound dressings within the context of precision regenerative surgery, emphasizing recent mechanistic discoveries, bioadaptive biomaterial strategies, and translational challenges shaping the future of wound therapeutics.

**Figure 1:**
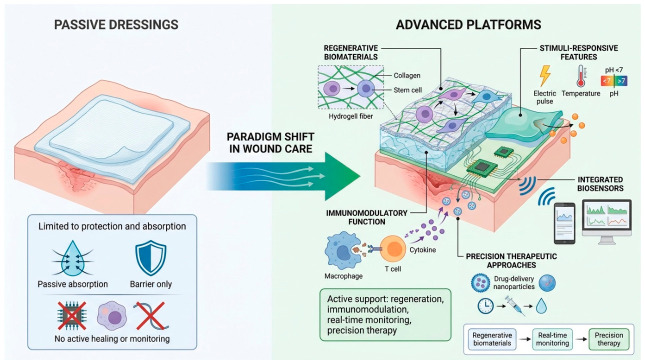
Evolution of advanced wound dressings. The figure illustrates the transition from conventional passive dressings toward bioactive, immunomodulatory, stimuli-responsive, and biosensor-integrated wound platforms. Emerging technologies increasingly combine regenerative biomaterials, real-time monitoring, and precision therapeutic approaches to actively support tissue repair and wound healing. Abbreviations: pH—potential of hydrogen. Created with www.BioRender.com by Urbanowicz T., ID: FL-PUB-20260620-MRGU3H).

The conceptual evolution of surgical wound dressings reflects a broader transition in regenerative medicine from passive tissue protection toward dynamic biological regulation of the wound microenvironment. Early wound dressings primarily functioned as mechanical barriers designed to provide moisture absorption and physical protection. However, advances in biomaterials science, immunology, nanotechnology, and bioelectronics have progressively transformed wound dressings into multifunctional therapeutic platforms capable of modulating inflammatory signaling, oxidative stress, angiogenesis, mechanotransduction, and regenerative cellular communication. As illustrated in Figure 1, contemporary wound engineering increasingly integrates adaptive biosensing, immunometabolic regulation, and precision-responsive therapeutic systems, reflecting the emergence of next-generation bioadaptive interfaces designed to actively direct tissue repair processes.

A central premise of this review is that the next major advance in surgical wound management will not arise from incremental improvement of occlusion, antimicrobial activity, or exudate control alone, but from programmable regulation of regenerative competence. In this framework, impaired wound healing is interpreted as a failure of coordinated biological control involving immune-cell plasticity, mitochondrial bioenergetics, redox balance, extracellular matrix mechanics, vascular adaptation, microbial ecology, and bioelectrical signaling. Advanced dressings should therefore be evaluated not simply as protective materials, but as regulatory interfaces capable of restoring the molecular, cellular, and biophysical conditions required for tissue regeneration. This perspective provides the conceptual basis for integrating immunometabolic reprogramming, bioadaptive biomaterials, intelligent biosensing, and precision-regenerative surgery into a unified therapeutic paradigm.

The aim of this review is to critically evaluate emerging advanced wound dressing technologies for surgical wound management, focusing on three converging areas: immunometabolic reprogramming, bioadaptive biomaterials, and intelligent regenerative interfaces. We discuss current mechanistic insights, translational evidence, clinical limitations, and future directions for precision regenerative wound care.

Although this review is framed around postoperative and precision surgical wound management, much of the mechanistic evidence derives from chronic wound models, including diabetic, ischemic, venous, and pressure wounds. These models do not fully reproduce acute surgical wounds; however, they provide relevant insight into persistent inflammation, impaired angiogenesis, mitochondrial dysfunction, microbial dysbiosis, senescence, and maladaptive mechanotransduction, which also contribute to complicated postoperative healing. Therefore, chronic wound evidence is interpreted here as a translational framework rather than direct clinical equivalence.

## 2. Immunobiology and Immunometabolism of Surgical Wound Healing

### 2.1. Beyond Classical Inflammation

The traditional classification of wound healing into hemostatic, inflammatory, proliferative, and remodeling phases inadequately reflects the biological complexity underlying tissue repair. Rather than a linear cascade, wound healing is now understood as a highly integrated and continuously adaptive process involving reciprocal communication among immune cells, stromal compartments, vascular networks, extracellular matrix structures, and metabolic signaling systems.

Immediately following surgical injury, platelet activation initiates coagulation cascades and provisional fibrin matrix formation while simultaneously releasing growth factors including platelet-derived growth factor (PDGF), transforming growth factor-β (TGF-β), epidermal growth factor (EGF), and vascular endothelial growth factor (VEGF) [23,24,25]. Neutrophils subsequently infiltrate the wound microenvironment and mediate antimicrobial defense through reactive oxygen species (ROS), neutrophil extracellular traps (NETs), and proteolytic enzymes [26,27,28]. However, excessive neutrophilic persistence contributes to collateral tissue injury and chronic inflammatory activation.

Macrophages are central regulators of wound healing [29,30]. Pro-inflammatory M1 macrophages dominate early inflammatory stages and mediate pathogen clearance by activating nitric oxide synthase, secreting tumor necrosis factor α (TNF-α), producing interleukin 1β (IL-1β), and generating ROS [31,32]. Transition toward reparative M2 phenotypes is subsequently required for angiogenesis, extracellular matrix deposition, fibroblast activation, and tissue remodeling [33,34,35]. Failure of appropriate macrophage polarization is increasingly recognized as a primary driver of chronic wound pathology.

Nevertheless, the classical M1/M2 macrophage polarization paradigm likely represents a substantial oversimplification of the highly dynamic immune heterogeneity observed within wound microenvironments [36]. Recent single-cell transcriptomic analyses have identified multiple intermediate and context-dependent macrophage activation states exhibiting overlapping inflammatory, reparative, metabolic, and angiogenic phenotypes that cannot be adequately classified within binary polarization frameworks [37,38]. These findings suggest that future immunomodulatory biomaterials may require more precise regulation of spatially and temporally evolving immune cell states rather than simply promoting generalized M2 polarization.

Importantly, macrophage function is closely linked to metabolic state. M1 macrophages rely predominantly on glycolytic metabolism, whereas M2 phenotypes favor oxidative phosphorylation and fatty acid oxidation. This emerging concept of immunometabolism has become central to contemporary wound biology.

### 2.2. Immunometabolic Reprogramming and Mitochondrial Dysfunction

Recent evidence suggests that metabolic dysregulation may play a more central role in impaired wound healing than previously appreciated [39,40,41]. Hypoxia, oxidative stress, ischemia–reperfusion injury, hyperglycemia, and mitochondrial dysfunction collectively contribute to pathological inflammatory persistence and regenerative failure.

Mitochondria function not only as bioenergetic organelles but also as critical regulators of innate immunity, apoptosis, ROS signaling, and inflammasome activation [42,43,44]. Excessive mitochondrial ROS production promotes endothelial dysfunction, fibroblast senescence, and chronic inflammatory signaling through nuclear factor kappa B (NF-κB) and NOD-, LRR-, and pyrin domain-containing protein 3 (NLRP3 inflammasome) activation [45,46]. Simultaneously, impaired oxidative phosphorylation reduces the availability of adenosine triphosphate (ATP), which is necessary for cellular migration, collagen synthesis, and angiogenesis [47,48].

Hypoxia-inducible factor-1α (HIF-1α), AMP-activated protein kinase (AMPK), mammalian target of rapamycin (mTOR), and PI3K/Akt signaling pathways have emerged as major regulators of wound immunometabolism [49,50,51,52,53,54,55]. Consequently, biomaterials capable of modulating local metabolic environments may represent highly promising therapeutic platforms.

Emerging hydrogel systems incorporating oxygen-generating nanoparticles, ROS-scavenging nanozymes, and mitochondrial-targeted antioxidants are increasingly being designed to restore metabolic homeostasis within ischemic or chronic wound microenvironments [56,57].

Accumulating evidence increasingly supports the concept that chronic wounds represent states of persistent immunometabolic dysfunction rather than isolated inflammatory lesions. Sustained glycolytic macrophage activation, mitochondrial fragmentation, ATP depletion, impaired oxidative phosphorylation, and excessive mitochondrial ROS production collectively generate a self-perpetuating pathological microenvironment characterized by angiogenic failure, fibroblast senescence, extracellular matrix degradation, and defective epithelial regeneration. Importantly, these findings suggest that future wound dressings may require the capacity to dynamically modulate cellular metabolism itself, including mitochondrial bioenergetics, redox equilibrium, and nutrient-sensitive signaling pathways, rather than functioning solely as anti-inflammatory or antimicrobial platforms.

To better contextualize the biological complexity underlying surgical wound repair, the major phases of wound healing and their associated molecular abnormalities are summarized in Table 1. Importantly, pathological wound healing is increasingly recognized as a consequence of dysregulated interactions among inflammatory signaling pathways, oxidative stress responses, extracellular matrix remodeling, mitochondrial dysfunction, and immune cell polarization. Understanding these mechanistic alterations is essential for the rational design of next-generation biomaterials capable of targeting specific pathological microenvironmental disturbances rather than functioning solely as passive wound coverings.

### 2.3. Hierarchical Control of Wound Regeneration

Wound repair may be conceptualized as a hierarchical biological control system in which successful tissue restoration depends on coordinated regulation across multiple interdependent levels. At the most basic level, wound dressings must provide structural protection, moisture balance, and microbial containment. At a second level, they should regulate excessive inflammation and oxidative injury. More advanced systems operate at the immunometabolic level by restoring macrophage plasticity, mitochondrial function, nutrient-sensitive signaling, and redox equilibrium. A further level involves control of mechanobiological and extracellular matrix cues, including stiffness, viscoelasticity, integrin signaling, Yes-associated protein and transcriptional coactivator with PDZ-binding motif (YAP/TAZ) activation, and fibroblast mechanical memory. At the highest level, regenerative competence requires integration of angiogenesis, epithelial migration, stromal remodeling, bioelectrical signaling, and resolution of pathological immune activation.

This hierarchical framework clarifies why many conventional and even bioactive dressings produce incomplete clinical benefit, as presented in Table 2. Materials that act only at the level of microbial control or passive protection may be insufficient in wounds dominated by mitochondrial dysfunction, persistent macrophage activation, senescence, ischemia, or maladaptive mechanotransduction. Conversely, next-generation bioadaptive dressings should be designed to intervene at several regulatory levels simultaneously. Such a model also enables more rational matching of biomaterial platforms to wound phenotypes, thereby prioritizing redox-responsive systems in oxidatively injured wounds, oxygen-generating matrices in ischemic wounds, immunomodulatory scaffolds in chronically inflamed wounds, and mechanoresponsive hydrogels in fibrotic or scar-prone surgical wounds.

## 3. Immuno-Instructive Biomaterials and Macrophage Reprogramming

One of the most important conceptual developments in advanced wound engineering involves the emergence of immuno-instructive biomaterials specifically designed to direct host immune responses toward regenerative phenotypes.

Unlike conventional dressings that passively isolate tissue, immunomodulatory biomaterials actively interact with immune cells through surface chemistry, mechanical properties, ionic release, ligand presentation, and degradation kinetics. These interactions profoundly influence macrophage polarization, inflammatory cytokine profiles, fibroblast behavior, and angiogenic signaling.

Surface nanotopography and matrix stiffness have particularly significant immunological consequences. Softer viscoelastic matrices often promote anti-inflammatory macrophage phenotypes, whereas rigid fibrotic microenvironments favor persistent inflammatory activation and fibroblast differentiation into contractile myofibroblasts. Consequently, mechanically adaptive biomaterials capable of dynamically modulating tissue stiffness have attracted substantial interest.

Recent advances in mechanoimmunology have further demonstrated that the mechanical properties of the extracellular matrix directly regulate innate immune programming and regenerative outcomes [81]. Matrix stiffness, viscoelasticity, cytoskeletal tension, and integrin-mediated mechanotransduction influence macrophage polarization, fibroblast activation, and angiogenic signaling through pathways involving YAP/TAZ, focal adhesion kinase, Piezo1, and nuclear mechanosensing systems [82,83,84]. Excessively rigid microenvironments promote persistent inflammatory activation and fibrotic remodeling, whereas mechanically adaptive matrices may facilitate regenerative immune phenotypes and scar attenuation [85,86]. Accordingly, future biomaterials may increasingly be engineered not only for biochemical functionality but also for precise control of mechanical signaling within the wound microenvironment.

Magnesium-based biomaterials, zwitterionic polymers, arginine-functionalized matrices, and (interleukin-4) IL-4-loaded hydrogels have all demonstrated the ability to promote M2 macrophage polarization while suppressing excessive TNF-α and IL-1β signaling [87,88]. Importantly, these systems may reduce pathological fibrosis while simultaneously enhancing angiogenesis and tissue remodeling.

The growing recognition that wound healing is fundamentally an immune-regulated process has shifted biomaterial design away from purely antimicrobial strategies toward restoration of immune homeostasis [89,90].

**Figure 2:**
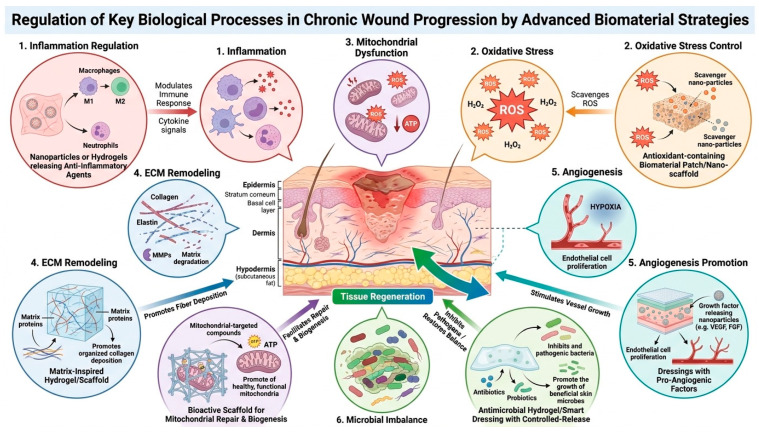
Immunometabolic drivers of chronic wound pathology and biomaterial-based therapeutic targets. The figure summarizes the major biological processes involved in chronic wound progression and the principal biomaterial strategies developed to regulate inflammation, oxidative stress, mitochondrial dysfunction, extracellular matrix remodeling, angiogenesis, and microbial imbalance, thereby supporting tissue regeneration. Abbreviations: ATP, adenosine triphosphate; ECM, extracellular matrix; MMPs, matrix metalloproteinases; ROS, reactive oxygen species. Created with www.BioRender.com by Urbanowicz T., ID: FL-PUB-20260620-TLMYU8).

Chronic wound pathology is increasingly recognized as a self-sustaining immunometabolic disorder driven by reciprocal interactions among persistent inflammation, mitochondrial dysfunction, oxidative stress, extracellular matrix degradation, fibroblast senescence, angiogenic failure, and microbial dysbiosis. Rather than functioning as isolated pathological events, these processes form highly interconnected regulatory networks that perpetuate regenerative failure and tissue instability. Importantly, contemporary biomaterial strategies are progressively designed to target multiple pathological nodes simultaneously through immunomodulation, redox regulation, metabolic reprogramming, extracellular matrix remodeling, and regenerative signaling delivery. As illustrated in Figure 2, advanced wound dressings are evolving toward systems-level therapeutic platforms that dynamically interact with the biological architecture of chronic wound microenvironments to restore tissue homeostasis and regenerative competence.

Because dysregulated inflammation represents a central driver of impaired tissue repair, increasing attention has focused on the development of biomaterials capable of actively modulating immune responses within the wound microenvironment. Table 3 summarizes the principal immunomodulatory strategies currently investigated in advanced wound engineering, highlighting their molecular targets, signaling pathways, and regenerative consequences. Importantly, these findings illustrate the ongoing paradigm shift from antimicrobial-centered wound management toward immune-regenerative therapeutic modulation.

**Figure 3:**
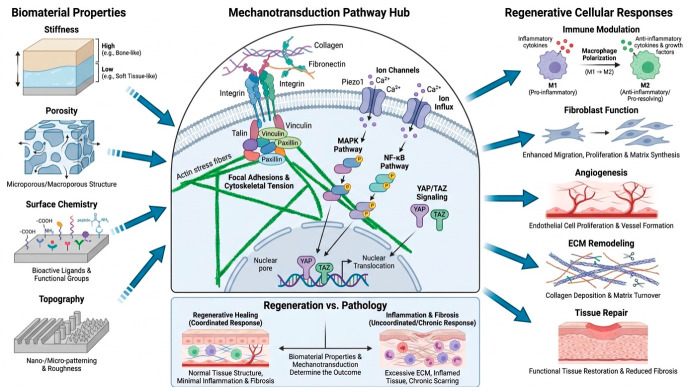
Interactions between biomaterial properties, mechanotransduction pathways, and regenerative cellular responses. The figure illustrates how biomaterial characteristics influence cellular signaling pathways that regulate immune responses, fibroblast behavior, angiogenesis, extracellular matrix remodeling, and tissue repair. Coordinated modulation of these processes may promote regenerative healing while reducing inflammation and fibrosis. Abbreviations: Ca^2+^, calcium ion; ECM, extracellular matrix; MAPK, mitogen-activated protein kinase; NF-κB, nuclear factor kappa B; YAP/TAZ, Yes-associated protein/transcriptional coactivator with PDZ-binding motif. Created with www.BioRender.com by Urbanowicz T., ID: FL-PUB-20260620-48XY31).

As illustrated in Figure 3, contemporary immuno-instructive biomaterials are increasingly engineered to integrate immunological, biochemical, and biomechanical cues in order to dynamically reprogram the wound microenvironment toward functional tissue regeneration.

Despite promising immunoregulatory effects in preclinical studies, precise control of macrophage polarization remains challenging, as excessive immunosuppression may impair antimicrobial defense and increase susceptibility to infection.

## 4. Smart Dressing Platforms as Bioadaptive Therapeutic Microenvironments

Hydrogels are among the most extensively investigated platforms in advanced wound engineering, owing to their structural resemblance to the native extracellular matrix and their exceptional physicochemical tunability [103,104,105].

However, contemporary hydrogel systems have evolved far beyond simple moisture-retaining materials. Modern hydrogels are increasingly designed as dynamic bioresponsive systems capable of immunoregulation [106,107], redox modulation [68,108,109], electrical conductivity [69,70], oxygen generation [71], stimuli-responsive drug release [72], and biosensor integration [73,74].

The rapid expansion of advanced biomaterials research has led to a diverse range of wound-dressing platforms with distinct structural, immunological, and regenerative properties. As summarized in Table 4, contemporary wound biomaterials increasingly integrate multifunctional capabilities including controlled therapeutic release, immunomodulation, redox regulation, electrical conductivity, and regenerative signaling. Comparative evaluation of these platforms is particularly important given the substantial differences in translational maturity, biological performance, manufacturing complexity, and clinical applicability among currently emerging systems.

### 4.1. Hydrogels as Dynamic Regenerative Matrices

Stimuli-responsive hydrogels constitute one of the most transformative developments in wound biomaterials research [75,76]. These systems dynamically respond to pathological wound-associated signals, including acidic pH, elevated ROS concentrations, hyperglycemia, inflammatory cytokines, protease overexpression, and hypoxia.

Responsive systems enable highly localized, temporally controlled therapeutic delivery while minimizing systemic toxicity.

ROS-responsive hydrogels [77,78,79] are particularly promising because oxidative stress represents a major contributor to endothelial dysfunction, fibroblast senescence, and chronic inflammation. However, complete elimination of ROS may impair physiological antimicrobial signaling and angiogenesis. Consequently, modern hydrogel systems increasingly aim to restore redox equilibrium rather than indiscriminately suppress oxidative pathways.

From a biomaterial design perspective, the key distinction lies between indiscriminate ROS elimination and redox modulation [80]. Full ROS-scavenging dressings may reduce oxidative injury but can also suppress ROS-dependent antimicrobial defense, angiogenic signaling, and cell migration. In contrast, ROS-responsive hydrogels, catalytic nanozymes, and oxygen-regulating matrices are designed to buffer excessive oxidative stress while preserving physiological redox signaling. This supports a homeostatic design strategy rather than maximal suppression of antioxidants. Representative redox-regulatory biomaterial strategies are summarized in Table 5.

As outlined in Table 6, these systems exploit wound-associated biochemical and biophysical abnormalities—including oxidative stress, hypoxia, protease overexpression, pH alterations, and hyperglycemia—to achieve highly localized and temporally regulated therapeutic delivery. Such bioresponsive platforms may substantially improve therapeutic precision while minimizing systemic toxicity and off-target biological effects.

Recent advances in systems biology, immunometabolism, mechanobiology, and regenerative medicine have identified several emerging molecular and cellular pathways that may represent highly promising therapeutic targets in next-generation wound engineering. As summarized in Table 7, these pathways extend beyond conventional antimicrobial and angiogenic strategies and increasingly involve regulation of ferroptosis, mitochondrial dysfunction, mechanotransduction, cellular senescence, inflammasome activation, trained immunity, and microbiome–host signaling interactions. Targeting these interconnected biological systems may substantially expand the therapeutic capabilities of future regenerative biomaterials.

Despite encouraging preclinical results, hydrogel systems face challenges, including batch-to-batch variability, sterilization requirements, limited mechanical durability, and a lack of high-quality randomized clinical trials. Most published studies remain confined to animal models, limiting assessment of long-term clinical effectiveness.

#### Antioxidant Paradox

Reactive oxygen species (ROS) occupy a paradoxical position in wound healing. While excessive ROS accumulation contributes to endothelial dysfunction, mitochondrial injury, inflammasome activation, fibroblast senescence, and extracellular matrix degradation, physiological ROS signaling remains essential for antimicrobial defense, angiogenesis, cell migration, and tissue regeneration. Consequently, complete elimination of ROS may be as detrimental as uncontrolled oxidative stress. This concept has important implications for biomaterial design. Early antioxidant wound dressings were largely developed to maximize ROS scavenging capacity; however, indiscriminate suppression of oxidative signaling may inadvertently impair normal regenerative processes and delay tissue repair. In contrast, contemporary redox-responsive biomaterials increasingly aim to modulate rather than abolish oxidative activity. For example, ROS-responsive hydrogels can selectively release therapeutic agents under conditions of excessive oxidative stress, whereas catalytic nanozyme systems based on cerium oxide or manganese dioxide continuously buffer pathological ROS while preserving physiological signaling thresholds. Such approaches seek to restore redox homeostasis rather than achieve complete neutralization of antioxidants. This shift from ROS elimination toward dynamic redox regulation reflects a broader transition in regenerative biomaterials from pathway inhibition to restoration of biological balance within the wound microenvironment. The principal strategies currently investigated for redox modulation in advanced wound dressings are summarized in Table 8.

### 4.2. Electrospun Nanofibers and Extracellular Matrix-Mimetic Scaffolds

Electrospinning technology has emerged as a highly versatile approach for fabricating biomimetic wound dressings that closely replicate the nanoscale architecture of the native extracellular matrix. Electrospun nanofibers provide high surface-area-to-volume ratios, interconnected porous networks, favorable oxygen diffusion, and excellent opportunities for incorporation of therapeutic cargo, including growth factors, extracellular vesicles, antimicrobial agents, nucleic acids, and bioactive nanoparticles.

Nanotechnology has transformed regenerative wound engineering by enabling precise spatiotemporal control over therapeutic delivery, cellular signaling, and antimicrobial activity.

Electrospun nanofibrous scaffolds closely mimic native collagen architecture and provide favorable microenvironments for fibroblast adhesion, endothelial migration, oxygen diffusion, and extracellular matrix organization [124,125]. Importantly, nanoscale structural properties—including fiber alignment, conductivity, stiffness gradients, and porosity—directly influence mechanotransduction pathways involving integrins, focal adhesion kinase (FAK), and YAP/TAZ signaling.

The integration of extracellular vesicles, antimicrobial peptides, conductive polymers, oxygen carriers, and RNA therapeutics within electrospun matrices has further transformed nanofibrous scaffolds into multifunctional regenerative interfaces.

Increasing evidence additionally suggests that chronic wound progression is strongly influenced by complex microbial ecosystem dynamics rather than simple bacterial contamination alone. Polymicrobial biofilms interact extensively with host immune pathways, metabolic signaling networks, and extracellular matrix remodeling processes, thereby contributing to persistent inflammation, antibiotic resistance, angiogenic impairment, and regenerative failure [126,127,131]. Importantly, conventional antimicrobial wound strategies frequently fail to adequately address these highly organized microbial communities. Consequently, emerging biomaterial platforms are increasingly being designed to modulate wound microbiome ecology through anti-biofilm nanoparticles, bacteriophage delivery systems, quorum-sensing interference, and microbiome-regulatory therapeutic approaches aimed at restoring microbial homeostasis rather than indiscriminately eliminating bacterial populations.

Recent developments have enabled the fabrication of multifunctional nanofibrous systems incorporating conductive polymers, oxygen-generating particles, extracellular vesicles, antimicrobial peptides, and immunomodulatory agents. Such hybrid platforms increasingly serve as bioactive regenerative interfaces that simultaneously provide structural support and biological regulation.

### 4.3. Nanozyme-Based Redox-Regulating Systems

Oxidative stress is a central pathological feature of chronic and impaired wound healing. Excessive reactive oxygen species (ROS) contribute to endothelial dysfunction, mitochondrial injury, inflammasome activation, fibroblast senescence, and extracellular matrix degradation. However, complete elimination of ROS may impair physiological antimicrobial defense, angiogenesis, and cellular signaling, creating what has been termed the “antioxidant paradox”.

Nanozymes [122,132] represent another rapidly emerging area. Unlike conventional antioxidants, nanozymes possess catalytic, enzyme-like activity that continuously scavenges ROS and modulates oxidative injury. Cerium oxide, platinum, manganese dioxide, and iron-based nanozymes exhibit superoxide dismutase- and catalase-like functions that may reduce chronic inflammatory damage while preserving physiological redox signaling.

Nevertheless, concerns regarding nanoparticle accumulation, biosafety, long-term toxicity, immune over-suppression, and biodegradation remain incompletely resolved and require careful translational investigation.

Despite their considerable therapeutic potential, nanomaterial-based wound systems remain associated with several unresolved biosafety concerns. Long-term nanoparticle accumulation, unpredictable biodegradation kinetics, macrophage overactivation, oxidative DNA injury, and potential systemic dissemination continue to represent important translational limitations. Furthermore, nanoscale physicochemical properties, including particle size, surface charge, morphology, and aggregation behavior, substantially influence cellular uptake and immunological responses. Consequently, future translational development of nanoengineered wound dressings will require rigorous long-term biosafety characterization together with standardized evaluation of nanomaterial–host interactions under clinically relevant conditions.

Contemporary nanozyme design increasingly emphasizes restoring redox balance rather than indiscriminately eliminating ROS. Redox-responsive nanozyme systems may therefore preserve physiological ROS-dependent regenerative signaling while preventing pathological oxidative injury. This approach better reflects the complex biological role of ROS during tissue repair and illustrates the broader transition from pathway inhibition toward homeostatic regulation in regenerative biomaterial engineering.

Long-term biodistribution, biodegradation, and nanotoxicity remain incompletely characterized. Regulatory pathways for nanozyme-based wound products are also not fully established.

### 4.4. Conductive Biomaterials and Bioelectrical Regeneration

Growing evidence indicates that endogenous bioelectrical signaling plays a critical role in tissue repair, influencing keratinocyte migration, angiogenesis, fibroblast activation, stem cell recruitment, and immune cell behavior. Tissue injury disrupts physiological electrical gradients, potentially contributing to delayed healing and impaired regeneration.

Beyond biochemical signaling, growing evidence demonstrates that biomaterial-mediated regulation of tissue repair is profoundly influenced by mechanical and biophysical interactions within the wound microenvironment. Matrix stiffness, viscoelasticity, nanotopography, electrical conductivity, and surface chemistry collectively regulate cellular behavior through highly integrated mechanotransduction pathways involving integrins, focal adhesion kinase (FAK), cytoskeletal remodeling, Piezo1 mechanosensors, calcium signaling, and YAP/TAZ-mediated nuclear mechanosensing [133,134]. These pathways directly influence macrophage polarization, fibroblast activation, endothelial migration, extracellular matrix organization, and stem cell fate determination, thereby governing the balance between regenerative healing and pathological fibrosis.

Conductive biomaterials, including graphene, MXenes, PEDOT:PSS, and polypyrrole, additionally facilitate restoration of endogenous bioelectrical signaling disrupted during tissue injury [135,136]. Bioelectrical stimulation has been shown to enhance keratinocyte migration, endothelial activation, angiogenesis, and mitochondrial ATP production.

The most advanced systems are now evolving toward closed-loop regenerative platforms in which biosensor feedback autonomously regulates therapeutic delivery. Such systems may ultimately enable responsive antibiotic release, adaptive oxygen generation, programmable anti-inflammatory signaling, and electrically stimulated angiogenesis.

Despite encouraging preclinical results, clinical translation remains limited by manufacturing complexity, long-term biocompatibility considerations, device integration challenges, and regulatory requirements. Nevertheless, conductive wound interfaces are increasingly regarded as an important component of future intelligent regenerative systems capable of integrating biosensing, therapeutic delivery, and adaptive bioelectrical stimulation.

## 5. Emerging Molecular Targets in Precision-Regenerative Wound Healing—Ferroptosis-Targeted Biomaterials

Ferroptosis, an iron-dependent form of lipid peroxidation-driven cell death, has recently emerged as a highly relevant mechanism in chronic wound pathology [137,138]. Excessive ferroptotic signaling contributes to endothelial injury, mitochondrial collapse, impaired angiogenesis, and inflammatory amplification.

Recent studies have demonstrated that matrix metalloproteinase (MMP)-responsive hydrogels incorporating ferroptosis inhibitors significantly improve angiogenesis and tissue regeneration in diabetic wound models [139]. These findings suggest that future wound dressings may increasingly function as metabolic and cell-death regulatory systems rather than merely structural biomaterials.

## 6. Extracellular Vesicle Engineering and Acellular Regenerative Therapies

Mesenchymal stem cell-derived extracellular vesicles (EVs), particularly exosomes, have emerged as highly promising acellular regenerative therapeutics [140,141]. These nanoscale vesicles contain bioactive cargo, including microRNAs, proteins, lipids, and signaling molecules that can recapitulate many of the therapeutic effects of parent stem cells.

Compared with live-cell transplantation, EV-based therapies offer several advantages [142,143], including reduced tumorigenic potential, improved biosafety, easier storage, lower immunogenicity, and greater translational scalability.

Recent studies [144,145,146,147] demonstrate that exosome-loaded hydrogels significantly enhance angiogenesis, collagen organization, re-epithelialization, and wound closure compared with exosomes or hydrogels alone. Current research directions increasingly focus on: programmable EV release, hypoxia-preconditioned exosomes, engineered exosomal cargo, and hybrid EV–hydrogel systems.

However, major translational challenges persist, including EV isolation standardization, cargo heterogeneity, rapid in vivo clearance, and lack of GMP-compliant manufacturing protocols.

Extracellular vesicle-based therapeutics have emerged as one of the most promising acellular regenerative strategies in modern wound engineering. By enabling targeted modulation of intercellular signaling without the limitations of live-cell transplantation, extracellular vesicles offer substantial translational potential for precision regenerative medicine. Table 9 summarizes the major extracellular vesicle sources, bioactive cargo profiles, biomaterial carriers, regenerative effects, and translational limitations currently associated with EV-based wound healing platforms.

Although extracellular vesicle-loaded dressings show substantial regenerative potential, standardization of EV isolation, potency testing, storage stability, and regulatory classification remains unresolved. Current evidence is largely preclinical, and robust human data remain scarce.

## 7. Bioelectronic and Intelligent Wound Interfaces

The integration of flexible electronics into wound dressings has generated a new generation of intelligent bioelectronic interfaces capable of real-time physiological monitoring and adaptive therapeutic response [158,159,160]. Modern bioelectronic dressings can continuously assess wound pH, oxygen saturation, glucose concentration, lactate levels, inflammatory cytokines, bacterial metabolites, and local temperature fluctuations.

Importantly, such parameters frequently change before overt clinical manifestations of infection or wound deterioration become apparent.

These developments represent a profound conceptual shift toward autonomous regenerative medicine.

**Figure 4:**
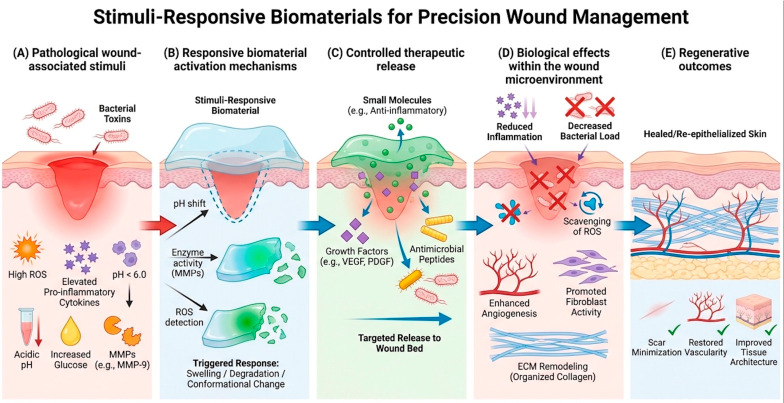
Stimuli-responsive biomaterials for precision wound management. The figure summarizes how adaptive biomaterial systems dynamically regulate wound healing in response to local microenvironmental changes. Abbreviations: AI, artificial intelligence; ATP, adenosine triphosphate; pH, potential of hydrogen; ROS, reactive oxygen species. Created with www.BioRender.com by Urbanowicz T., ID: FL-PUB-20260620-4IO4B0).

The convergence of biomaterials science, flexible bioelectronics, biosensor engineering, wireless communication technologies, and artificial intelligence has enabled the emergence of highly sophisticated wound interfaces that continuously monitor and dynamically regulate the wound microenvironment. Unlike conventional dressings that provide static therapeutic support, next-generation bioadaptive systems increasingly serve as closed-loop regenerative platforms that integrate real-time biochemical sensing with responsive therapeutic delivery. These systems may detect pathological alterations in pH, oxidative stress, oxygen tension, inflammatory cytokines, glucose concentration, bacterial metabolites, and exudate composition before overt clinical deterioration becomes apparent. As illustrated in Figure 4, intelligent wound dressings are progressively evolving toward autonomous therapeutic interfaces capable of adaptive immunomodulation, precision drug delivery, oxygen generation, anti-biofilm regulation, and personalized regenerative control through continuous feedback-guided wound management.

## 8. Mechanobiology, Senescence, and Emerging Frontiers

Mechanobiological signaling has emerged as a major determinant of wound fibrosis, regeneration, and scar quality [161,162]. Matrix stiffness, viscoelasticity, and cellular traction forces directly regulate fibroblast differentiation, macrophage activation, and angiogenic signaling through mechanotransduction pathways including YAP/TAZ, integrin signaling, and focal adhesion kinase activation.

Simultaneously, cellular senescence has become increasingly recognized as a major driver of chronic wound pathology. Senescent fibroblasts and endothelial cells exhibit senescence-associated secretory phenotypes (SASP) characterized by chronic inflammatory cytokine release, protease overexpression, and impaired regenerative capacity [163,164,165].

Recent evidence increasingly suggests that cellular senescence represents not merely a consequence of impaired wound healing, but an active pathogenic driver of chronic regenerative failure. Senescent cells accumulate within chronic wound environments and secrete complex senescence-associated secretory phenotypes (SASP) characterized by persistent inflammatory cytokines, matrix-degrading proteases, oxidative mediators, and pro-fibrotic signaling molecules. These factors amplify immune dysregulation, impair stem cell function, and perpetuate extracellular matrix degradation. Consequently, senolytic biomaterials and senescence-modulating therapeutic platforms may emerge as highly promising strategies for restoration of regenerative competence in chronic and postoperative wounds.

Accordingly, senolytic biomaterials and mechanoresponsive hydrogels capable of regulating fibroblast mechanical memory may represent highly promising avenues for the future.

Additional emerging strategies include microbiome-engineered wound therapeutics, bacteriophage-loaded dressings, mitochondrial transfer technologies, clustered regularly interspaced short palindromic repeats (CRISPR)-enabled regenerative biomaterials, and AI-guided predictive wound analytics.

### Biological Controversies and Unresolved Questions

Several major biological uncertainties remain unresolved and should temper the interpretation of preclinical success. First, although macrophage polarization is frequently described using the M1/M2 framework, this binary model does not adequately capture the dynamic, tissue-specific, and metabolically heterogeneous immune states observed during wound repair. Biomaterials designed merely to “promote M2 polarization” may therefore oversimplify the immune requirements for functional regeneration. The therapeutic objective should be immune-state resolution and regenerative coordination rather than indiscriminate induction of a single macrophage phenotype.

Second, the causal role of ferroptosis, senescence, and mitochondrial dysfunction in impaired wound healing remains incompletely defined. These mechanisms are strongly associated with chronic and metabolically compromised wounds, but their relative contributions may vary substantially depending on ischemia, diabetes, infection, age, and surgical context. Third, excessive antioxidant or anti-inflammatory activity may paradoxically impair normal healing because moderate ROS generation and transient inflammation are required for antimicrobial defense, angiogenic signaling, and cellular recruitment. These considerations indicate that next-generation dressings should restore biological balance rather than globally suppress individual pathways.

Finally, the clinical significance of highly sophisticated technologies such as extracellular vesicle-loaded hydrogels, nanozyme dressings, and intelligent bioelectronic interfaces remains to be established in rigorous human studies. Many platforms show impressive effects in small-animal models, yet translation is constrained by species-specific healing mechanisms, limited reproducibility, manufacturing complexity, cost, and uncertain regulatory classification. The field, therefore, requires a shift from proof-of-concept innovation toward comparative effectiveness, standardized potency testing, and clinically meaningful outcome validation.

## 9. Clinical Translation and Current Evidence

Despite substantial advances in biomaterial engineering, translation of advanced wound dressings into routine clinical practice remains limited. Most evidence derives from preclinical studies, while human data are largely confined to early-phase clinical investigations.

### 9.1. Existing Clinical Evidence

A relatively few advanced wound-dressing systems have successfully progressed to robust clinical implementation. Current human evidence remains dominated by early-phase trials, pilot translational studies, and small-cohort investigations rather than large, multicenter, randomized studies [166,167,168,169]. Nevertheless, several advanced biomaterial platforms have demonstrated encouraging therapeutic outcomes in chronic wounds, diabetic ulcers, ischemic injuries, and postoperative wound management. Table 10 summarizes representative clinical and translational studies investigating emerging wound-dressing technologies, along with their major therapeutic outcomes, translational limitations, and current stages of clinical development.

Although advanced wound dressings have shown substantial mechanistic promise, their clinical translation remains uneven. Most human evidence is still derived from small, heterogeneous studies with variable wound etiologies, inconsistent endpoints, short follow-up, and limited mechanistic biomarker integration. Moreover, many platforms demonstrate superiority in preclinical models but fail to provide robust comparative data against optimized standard care in real-world surgical populations. Cost-effectiveness, reimbursement feasibility, sterilization stability, batch-to-batch reproducibility, long-term biosafety, and regulatory classification remain major barriers to implementation. These limitations are particularly relevant for nanozyme-based systems, extracellular vesicle-loaded scaffolds, smart bioelectronic dressings, and oxygen-generating matrices, where clinical validation remains substantially less mature than preclinical development. Therefore, future trials should prioritize multicenter randomized designs, standardized wound-healing endpoints, patient stratification, mechanistic biomarkers, health-economic analyses, and longer follow-up to determine whether bioadaptive wound platforms provide clinically meaningful advantages beyond conventional advanced dressings.

### 9.2. Translational Barriers

Although accumulating clinical evidence supports the therapeutic potential of several advanced wound dressing platforms, translation from experimental systems to routine clinical practice remains challenging. Many technologies that demonstrate promising regenerative effects in preclinical models fail to achieve widespread adoption due to limitations related to manufacturing, reproducibility, cost, scalability, and clinical validation. Moreover, substantial heterogeneity among wound types, patient populations, biomaterial compositions, and outcome measures complicates direct comparison between studies. Understanding these translational barriers is essential for a realistic assessment of the future clinical impact of advanced regenerative dressings.

### 9.3. Regulatory Challenges

Beyond scientific and technological limitations, regulatory considerations represent a major determinant of successful clinical implementation. Contemporary wound dressings increasingly combine biomaterials, biologically active agents, extracellular vesicles, biosensors, and digital monitoring systems, creating products that may not fit easily within traditional regulatory frameworks. The resulting complexity affects product classification, safety evaluation, manufacturing requirements, quality control, and approval pathways. Consequently, regulatory challenges have emerged as one of the principal obstacles to the translation of next-generation wound technologies from laboratory research into clinical practice.

### 9.4. Future Clinical Trials

Addressing both translational and regulatory limitations will require a new generation of well-designed clinical investigations. While numerous preclinical studies have demonstrated encouraging biological effects, high-quality human evidence remains comparatively limited for many advanced wound platforms. Future clinical trials should therefore move beyond simple wound-closure endpoints and incorporate mechanistic biomarkers, patient stratification strategies, health-economic analyses, and long-term regenerative outcomes. Such studies will be critical for determining which technologies provide meaningful clinical benefit and for establishing evidence-based frameworks for precision wound management.

## 10. Current Controversies and Unresolved Challenges

Despite extraordinary progress in regenerative biomaterials research, several fundamental biological and translational controversies remain unresolved. Although macrophage-directed immunomodulation is widely regarded as therapeutically beneficial, excessive promotion of reparative M2 phenotypes may paradoxically contribute to pathological fibrosis and aberrant extracellular matrix deposition. Similarly, while antioxidant biomaterials effectively reduce oxidative injury, excessive ROS suppression may impair physiological antimicrobial defense and angiogenic signaling required for effective tissue repair. Extracellular vesicle-based therapies additionally face major challenges associated with cargo heterogeneity, donor variability, rapid in vivo clearance, and lack of standardized isolation protocols. Conductive bioelectronic dressings introduce further concerns regarding long-term biocompatibility, device integration stability, signal drift, and potential immune activation associated with chronic electronic interfaces. Moreover, substantial discrepancies between preclinical animal models and human wound physiology continue to limit translational reproducibility. Collectively, these unresolved issues highlight the need for more mechanistically integrated and clinically standardized approaches in future wound biomaterials research.

Ultimately, the future of surgical wound dressings may lie not in the development of increasingly complex wound coverings, but in the emergence of adaptive immunometabolic interfaces capable of dynamically regulating the biological architecture of tissue regeneration itself.

**Figure 5:**
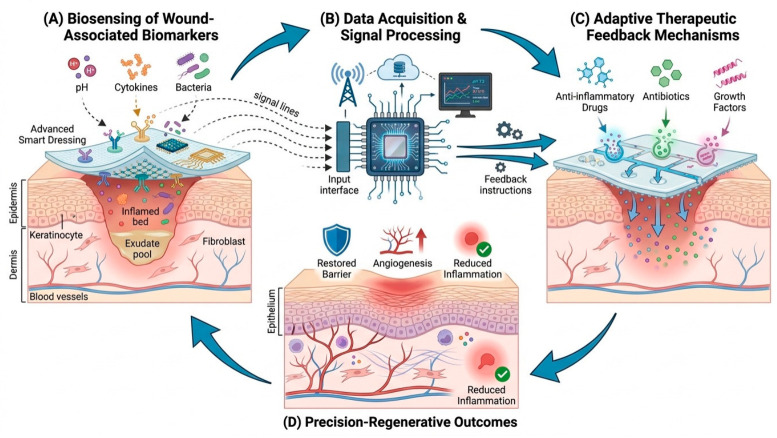
Components of intelligent wound-management systems. The figure summarizes the concept of closed-loop wound management, which relies on real-time monitoring and individualized therapeutic regulation. Abbreviation: pH, potential of hydrogen. Created with www.BioRender.com by Urbanowicz T., ID: FL-PUB-20260620-6Q7GA7).

The future trajectory of regenerative wound engineering is increasingly moving beyond isolated biomaterial innovation toward integrated precision-regenerative ecosystems capable of simultaneously modulating immune signaling, metabolism, mechanobiology, microbial ecology, vascular function, and tissue-specific regenerative responses across multiple biological scales. Emerging advances in spatial multi-omics, bioelectronics, artificial intelligence, programmable biomaterials, extracellular vesicle engineering, robotic wound management, and regenerative biofabrication collectively suggest that future wound therapeutics may evolve into highly adaptive systems capable of continuously sensing, predicting, and dynamically regulating tissue repair processes in real time. As illustrated in Figure 5, next-generation wound healing strategies are expected to increasingly integrate systems biology, computational medicine, bioengineering, and precision therapeutics into coordinated regenerative platforms designed not only to accelerate wound closure but to restore functional tissue architecture and long-term tissue homeostasis.

## 11. Translational Relevance of Chronic Wound Models to Surgical Wound Healing

Contemporary wound management strategies increasingly recognize that impaired tissue repair represents a biologically heterogeneous process strongly influenced by patient-specific immunological, metabolic, vascular, microbiological, and mechanical factors. Although many mechanistic insights discussed in this review originate from studies performed in diabetic, venous, and pressure ulcer models, several biological processes—including macrophage polarization, oxidative stress, mitochondrial dysfunction, mechanotransduction, angiogenesis, and extracellular matrix remodeling—are shared across chronic and postoperative wound environments. Nevertheless, important differences exist. Surgical wounds are typically acute injuries characterized by predictable healing trajectories and lower microbial burden, whereas chronic wounds are marked by persistent inflammation, ischemia, senescence, and biofilm formation. Consequently, direct translation of chronic wound biomaterial strategies into surgical wound management requires careful clinical validation.

Accordingly, next-generation regenerative biomaterials are increasingly being designed as precision-responsive therapeutic systems integrating biomarker-guided drug delivery, adaptive biosensing, programmable immunomodulation, and real-time physiological monitoring. Artificial intelligence-assisted wound analytics, predictive computational modeling, and digital wound phenotyping may additionally enable dynamic adjustment of therapeutic strategies according to evolving wound biology during the healing process. Collectively, these developments suggest that the future of surgical wound management may increasingly depend on the integration of regenerative biomaterials with personalized systems medicine approaches capable of continuously interpreting and modulating patient-specific tissue repair responses.

Based on the presented data, we propose biomarker-guided selection of advanced wound dressing different strategies as presented in Table 11.

## 12. Translational Readiness of Advanced Wound Dressing Technologies

A major limitation of the current wound biomaterials field is the imbalance between experimental innovation and translational maturity. Hydrogels, antimicrobial dressings, and selected extracellular matrix-based platforms have achieved partial clinical adoption because their manufacturing, sterilization, storage, and regulatory pathways are comparatively well established. In contrast, extracellular vesicle systems, nanozyme-based dressings, ferroptosis-targeted biomaterials, and closed-loop bioelectronic interfaces remain at earlier stages of development despite strong mechanistic appeal. Their clinical translation requires not only demonstration of biological efficacy but also reproducible manufacturing, batch-release criteria, long-term biosafety data, cost-effectiveness, and clear regulatory classification.

A technology-readiness perspective is therefore essential when evaluating the future impact of advanced wound dressings. Platforms that appear mechanistically sophisticated may not necessarily be closest to clinical implementation, whereas simpler biomaterials with robust manufacturability and clear endpoints may reach patients sooner. The most clinically successful systems are likely to be those that combine biological specificity with practical feasibility, including ease of use in surgical workflows, compatibility with sterilization, stability during storage, affordability, and measurable superiority over existing standards of care.

Although numerous advanced wound-dressing technologies have demonstrated promising regenerative and immunomodulatory effects in preclinical studies, their translation into routine clinical practice remains highly variable. Differences in manufacturing complexity, regulatory requirements, scalability, biosafety profiles, and the availability of clinical evidence continue to influence the readiness of individual platforms for clinical adoption. The current translational status of major emerging wound-dressing technologies, together with the principal barriers limiting their implementation, is summarized in Table 12.

## 13. Translational Challenges and Clinical Perspectives

Despite extraordinary scientific progress, major translational barriers continue to limit widespread clinical implementation of advanced wound dressings.

Importantly, a major limitation in regenerative wound engineering involves the persistent discrepancy between highly promising preclinical outcomes and comparatively modest clinical translation success. Many advanced biomaterial platforms demonstrate substantial regenerative efficacy in controlled experimental models yet fail to reproduce equivalent therapeutic benefit in clinically heterogeneous human wounds characterized by complex systemic comorbidities, polymicrobial colonization, impaired vascularization, metabolic dysregulation, and prolonged inflammatory activation. Furthermore, substantial biological differences between murine and human wound healing—including the dominant role of wound contraction in rodent models versus re-epithelialization in human tissue repair—continue to limit translational predictability. These discrepancies highlight the urgent need for clinically relevant experimental models capable of more accurately recapitulating human wound pathophysiology. Despite considerable progress in advanced wound biomaterials, several important barriers continue to limit their widespread clinical adoption. These include a lack of large multicenter randomized clinical trials, challenges related to scalable manufacturing and sterilization, unresolved long-term biosafety concerns, complex regulatory approval pathways, and uncertainties regarding cost-effectiveness in routine clinical practice.

Combination products integrating biomaterials, biologics, nanoparticles, and bioelectronics face particularly challenging approval pathways. Additionally, discrepancies between preclinical animal models and human wound physiology frequently hinder translational reproducibility. Future advances in regenerative wound care will depend on the establishment of standardized biomaterial characterization protocols, implementation of Good Manufacturing Practice (GMP)-compliant production processes, comprehensive long-term biosafety assessment, and the design of robust clinically relevant trials capable of demonstrating therapeutic efficacy and translational value. Finally, we propose a novel roadmap for the next decade of precision-regenerative wound dressing development, as presented in Table 13.

Most advanced wound dressing platforms remain supported primarily by preclinical evidence. Major limitations include limited randomized human trials, lack of standardized wound phenotyping, heterogeneity of animal models, insufficient head-to-head comparisons between biomaterial classes, incomplete long-term biosafety data for nanomaterials and bioelectronics, and unclear regulatory pathways for combination products. Future studies should prioritize clinically meaningful endpoints, biomarker-guided patient selection, standardized potency assays, and direct comparison with established wound-care standards.

## 14. Conclusions

The field of wound dressings is undergoing a profound transformation. Materials originally developed as passive barriers are increasingly engineered to actively participate in tissue repair by modulating inflammation, cellular metabolism, angiogenesis, extracellular matrix remodeling, and host–microenvironment interactions. Advances in biomaterials science, immunology, nanotechnology, bioelectronics, and regenerative medicine have generated a broad spectrum of innovative therapeutic platforms with the potential to improve surgical wound management.

At the same time, our understanding of wound healing has evolved considerably. Impaired healing is now recognized as a consequence of complex disturbances involving immune regulation, mitochondrial function, oxidative stress, mechanotransduction, cellular senescence, and microbial ecology. Consequently, the therapeutic objective is shifting from simple infection control or moisture maintenance toward restoration of a regenerative microenvironment capable of supporting coordinated tissue repair. In this context, immunomodulatory biomaterials, stimuli-responsive hydrogels, extracellular vesicle-based systems, nanozyme platforms, and intelligent bioelectronic dressings represent particularly promising directions.

Despite these advances, the distance between experimental innovation and routine clinical application remains substantial. Most emerging technologies continue to rely on preclinical evidence, while issues related to manufacturing, standardization, regulatory approval, long-term safety, and cost-effectiveness remain incompletely resolved. Future progress will depend not only on the development of increasingly sophisticated biomaterials but also on rigorous clinical validation and a better understanding of which patients are most likely to benefit from specific therapeutic approaches.

Ultimately, the next generation of wound dressings will likely be defined not by their physical properties alone, but by their ability to interact dynamically with the biological processes that govern tissue regeneration. Whether these technologies can translate into meaningful improvements in surgical outcomes remains one of the most important challenges for regenerative medicine in the coming decade.

## Figures and Tables

**Table 1:** Biological Phases of Surgical Wound Healing and Associated Molecular Dysregulation.

Wound Healing Phase	Dominant Cell Types	Major Molecular Mediators	Pathological Dysregulation	Potential Biomaterial Targets	References
Hemostasis	Platelets, endothelial cells	PDGF, TGF-β, thrombin, fibrin	Excessive coagulation, impaired clot stability	Hemostatic hydrogels, fibrin-based matrices	[4,58,59]
Inflammatory phase	Neutrophils, M1 macrophages	TNF-α, IL-1β, IL-6, ROS, NF-κB	Persistent inflammation, excessive ROS, inflammasome activation	ROS-responsive hydrogels, immunomodulatory biomaterials	[2,60,61]
Proliferative phase	Fibroblasts, endothelial cells, keratinocytes	VEGF, FGF, HIF-1α, collagen, MMPs	Impaired angiogenesis, fibroblast senescence, ECM degradation	Pro-angiogenic scaffolds, oxygen-generating dressings	[62,63]
Remodeling phase	Myofibroblasts, M2 macrophages	TGF-β, collagen I/III, TIMPs	Fibrosis, hypertrophic scarring, chronic matrix remodeling	Mechanoresponsive biomaterials, anti-fibrotic hydrogels	[64,65]
Chronic pathological wound state	Senescent fibroblasts, persistent M1 macrophages	ROS, SASP cytokines, MMP overexpression, NLRP3 inflammasome	Chronic inflammation, biofilm persistence, mitochondrial dysfunction	Senolytic biomaterials, nanozymes, EV-loaded dressings	[66,67]

Abbreviations: ECM—Extracellular Matrix, EV—Extracellular Vesicle, FGF—Fibroblast Growth Factor, HIF-1α—Hypoxia-Inducible Factor 1-alpha, IL-1β—Interleukin-1 beta, IL-6—Interleukin-6, MMPs—Matrix Metalloproteinases, NF-κB—Nuclear Factor kappa B, NLRP3—NOD-, LRR- and pyrin domain-containing protein 3, PDGF—Platelet-Derived Growth Factor, ROS—Reactive Oxygen Species, SASP—Senescence-Associated Secretory Phenotype, TGF-β—Transforming Growth Factor beta, TIMPs—Tissue Inhibitors of Metalloproteinases, TNF-α—Tumor Necrosis Factor alpha, VEGF—Vascular Endothelial Growth Factor.

**Table 2:** Hierarchical regulatory model of advanced wound dressings.

Regulatory Level	Dominant Biological Problem	Dressing Function	Representative Biomaterial Strategy	Translational Implication	References
Level 1: Protection	Contamination, fluid loss, mechanical trauma	Barrier function and moisture control	Gauze, films, hydrocolloids, foams	Clinically established but biologically passive	[15,16]
Level 2: Antimicrobial control	Bacterial burden, early infection risk	Local antimicrobial activity	Silver, iodine, PHMB, antibiotic-loaded matrices	Useful but insufficient for complex chronic wounds	[11,17,18]
Level 3: Inflammatory control	Persistent neutrophil/macrophage activation, cytokine excess	Immune modulation and ROS balancing	IL-4 hydrogels, ROS-responsive hydrogels, anti-inflammatory matrices	Requires careful avoidance of immune over-suppression	[29,30,31,32,33,34,35,36,37,38,62,63,64,65]
Level 4: Immunometabolic repair	Mitochondrial dysfunction, glycolytic immune persistence, ATP depletion	Restore metabolic homeostasis	Oxygen-generating matrices, mitochondrial antioxidants, nanozymes	Strong mechanistic rationale; limited human validation	[39,40,41,42,43,44,45,46,47,48,49,50,51,52,53,54,55,56,57]
Level 5: Mechanobiological regulation	Fibrosis, abnormal stiffness, fibroblast mechanical memory	Control ECM mechanics and mechanotransduction	Mechanoresponsive hydrogels, viscoelastic scaffolds, conductive matrices	Important for surgical scars and fibrotic wounds	[2,4,58,59,60,61,66,67]
Level 6: Regenerative integration	Failed angiogenesis, epithelialization, stromal remodeling	Coordinate tissue regeneration	EV-loaded scaffolds, growth-factor systems, bioelectronic platforms	Promising but requires potency assays and clinical endpoints	[68,69,70,71,72,73,74,75,76,77,78,79,80]
Level 7: Adaptive precision control	Dynamic wound heterogeneity	Real-time sensing and therapeutic adjustment	Biosensor-integrated smart dressings, AI-guided closed-loop systems	Future direction; regulatory and cost barriers remain high	[76,77,78,79,80]

Abbreviations: AI, Artificial Intelligence; ATP, Adenosine Triphosphate; ECM, Extracellular Matrix; EV, Extracellular Vesicle; IL-4, Interleukin-4; PHMB, Polyhexamethylene Biguanide; ROS, Reactive Oxygen Species.

**Table 3:** Immunomodulatory Mechanisms of Advanced Wound Dressings.

Biomaterial Strategy	Immune Target	Molecular Pathway	Regenerative Outcome	References
IL-4-loaded hydrogels	M2 macrophage polarization	STAT6 activation	Reduced inflammation and enhanced angiogenesis	[91,92]
ROS-scavenging nanozymes	Oxidative stress modulation	Nrf2 pathway activation	Reduced endothelial injury and fibroblast senescence	[93]
Magnesium-based biomaterials	Macrophage phenotype regulation	PI3K/Akt signaling	Enhanced tissue remodeling	[94,95]
Zwitterionic polymers	Immune cell adhesion reduction	NF-κB suppression	Reduced chronic inflammatory activation	[96,97]
Exosome-loaded scaffolds	Immune microenvironment modulation	miRNA-mediated signaling	Accelerated wound closure and angiogenesis	[98,99]
Conductive biomaterials	Bioelectric immune regulation	Calcium signaling/YAP-TAZ	Improved epithelial migration	[59,100]
Ferroptosis-targeted hydrogels	Lipid peroxidation inhibition	GPX4 stabilization	Enhanced endothelial survival and vascularization	[101,102]

Abbreviations: IL-4—Interleukin-4, ROS—Reactive Oxygen Species, STAT6—Signal Transducer and Activator of Transcription 6, Nrf2—Nuclear Factor Erythroid 2–Related Factor 2, PI3K—Phosphoinositide 3-Kinase, Akt—Protein Kinase B, NF-κB—Nuclear Factor Kappa B, miRNA—MicroRNA, YAP/TAZ—Yes-Associated Protein/Transcriptional Coactivator with PDZ-Binding Motif, GPX4—Glutathione Peroxidase 4, M2 macrophages—Alternatively Activated (Anti-inflammatory) Macrophages.

**Table 4:** Advanced Biomaterial Platforms in Surgical Wound Healing.

Biomaterial Platform	Principal Mechanism	Biological Target	Major Advantages	Main Limitations	Translational Stage	References
Hydrogels	Moisture retention, controlled therapeutic release	ECM mimicry, inflammation modulation	High biocompatibility, tunable properties	Mechanical instability, degradation variability	Clinically established/advanced translational	[68,69,70,71,72,73,74,103,104,105,106,107,108,109]
Electrospun nanofibers	ECM structural mimicry	Cell migration, angiogenesis	High surface area, biomolecule loading capacity	Sterilization and scalability challenges	Translational/preclinical	[80,110,111,112,113]
Nanozyme-based dressings	Catalytic ROS scavenging	Oxidative stress, mitochondrial dysfunction	Sustained antioxidant activity	Nanotoxicity concerns	Experimental	[114,115]
Conductive biomaterials	Restoration of bioelectrical signaling	Angiogenesis, keratinocyte migration	Electrical stimulation of regeneration	Bioelectronic integration complexity	Early translational	[116,117,118,119]
Oxygen-generating matrices	Local oxygen release	Tissue hypoxia, ischemia	Enhanced angiogenesis and ATP production	Oxygen-release control difficulty	Experimental	[56,57,71]
EV-loaded biomaterials	Intercellular regenerative signaling	Angiogenesis, immune regulation	Acellular regenerative therapy	EV isolation standardization	Preclinical/translational	[120,121,122,123,124,125,126,127]
Smart bioelectronic dressings	Real-time biosensing and adaptive response	Infection monitoring, metabolic regulation	Personalized wound management	Signal drift, cost, regulatory barriers	Early clinical development	[73]

Abbreviations: ATP—Adenosine Triphosphate, ECM—Extracellular Matrix, EV—Extracellular Vesicle, ROS—Reactive Oxygen Species.

**Table 5:** Comparative Approaches to Redox Homeostasis in Advanced Wound Dressings.

Therapeutic Strategy	Biological Rationale	Effect on ROS Signaling	Representative Biomaterial Platform	Potential Limitation	Therapeutic Strategy	References
Non-selective antioxidant delivery	Broad reduction of oxidative stress	Suppresses both physiological and pathological ROS	Conventional antioxidant-loaded dressings	May impair angiogenesis, antimicrobial defense, and regenerative signaling	Non-selective antioxidant delivery	[110]
ROS-responsive hydrogels	Activation only under excessive oxidative stress	Preserves physiological ROS while limiting oxidative injury	Thioketal-linked or ROS-cleavable hydrogels	Dependent on local ROS threshold accuracy	ROS-responsive hydrogels	[111]
Catalytic nanozyme systems	Continuous redox buffering through enzyme-mimetic activity	Dynamic modulation of ROS homeostasis	Cerium oxide, manganese dioxide, platinum nanozymes	Long-term biosafety and biodegradation concerns	Catalytic nanozyme systems	[112]
Oxygen-generating biomaterials	Reduction of hypoxia-associated oxidative dysfunction	Indirect restoration of redox balance	Peroxide-based oxygen-releasing matrices	Difficult control of oxygen-release kinetics	Oxygen-generating biomaterials	[113]
Mitochondria-targeted antioxidant platforms	Protection of mitochondrial bioenergetics and signaling	Reduction of mitochondrial ROS generation	Mitochondria-targeted nanoparticles and hydrogels	Limited clinical validation	Mitochondria-targeted antioxidant platforms	[114,115]
Immunometabolic biomaterials	Restoration of immune-cell metabolic homeostasis	Secondary normalization of ROS production	Immunomodulatory hydrogels and macrophage-reprogramming matrices	Complex biological response variability	Immunometabolic biomaterials	[116,117]

Abbreviations: ROS—Reactive Oxygen Species.

**Table 6:** Stimuli-Responsive Smart Dressings and Their Therapeutic Functions.

Stimulus	Responsive Biomaterial	Triggered Therapeutic Action	Clinical Purpose
Acidic pH	pH-responsive hydrogel	Antibiotic release	Infection control
Elevated ROS	ROS-sensitive nanozyme hydrogel	Antioxidant release	Redox homeostasis restoration
Hyperglycemia	Glucose-responsive matrix	Insulin or drug release	Diabetic wound management
Protease overexpression	MMP-sensitive hydrogel	Growth factor liberation	Chronic wound remodeling
Hypoxia	Oxygen-generating scaffold	Sustained oxygen release	Angiogenesis enhancement
Temperature elevation	Thermoresponsive polymer	Anti-inflammatory drug delivery	Early infection management
Electrical stimulation	Conductive hydrogel	Bioelectrical activation	Enhanced tissue regeneration

Abbreviations: MMP—Matrix Metalloproteinase, ROS—Reactive Oxygen Species.

**Table 7:** Emerging Biological Targets in Precision-Regenerative Wound Healing.

Emerging Target	Biological Role	Biomaterial Strategy	Regenerative Potential	References
Ferroptosis	Lipid peroxidation-mediated cell death	Ferroptosis-inhibiting hydrogels	Endothelial protection and angiogenesis	[128,129,130]
Mitochondrial dysfunction	ATP depletion and oxidative injury	Mitochondria-targeted antioxidants	Restoration of metabolic homeostasis	[42,43,44,45,46,47,48,49,50,51,52,53,54,55,56,57]
YAP/TAZ signaling	Mechanotransduction and fibrosis	Mechanoresponsive biomaterials	Scar attenuation and regenerative repair	[81,82,83,84,85,86,116,117]
Cellular senescence	SASP-mediated chronic inflammation	Senolytic biomaterials	Restoration of regenerative competence	[45,46,66,67]
NLRP3 inflammasome	Chronic inflammatory activation	ROS-responsive nanozymes	Immune regulation and reduced tissue injury	[2,45,46,60,61]
Trained immunity	Persistent innate immune memory	Immunomodulatory scaffolds	Adaptive inflammatory regulation	[89,90]
Biofilm–microbiome signaling	Chronic microbial persistence	Anti-biofilm biomaterials	Restoration of microbial homeostasis	[111,112,113]
Hypoxia signaling	Angiogenic regulation	Oxygen-generating matrices	Improved vascular regeneration	[49,50,51,52,53,54,55,71]

Abbreviations: ATP—Adenosine Triphosphate, NLRP3—NOD-, LRR-, and Pyrin Domain-Containing Protein 3, ROS—Reactive Oxygen Species, SASP—Senescence-Associated Secretory Phenotype, YAP/TAZ—Yes-Associated Protein/Transcriptional Coactivator with PDZ-Binding Motif.

**Table 8:** Redox-Regulatory Biomaterial Strategies in Advanced Wound Dressings.

Strategy	Effect	References
Full ROS scavenging	Reduces oxidative injury but may impair angiogenesis and antimicrobial defense	[118,119]
ROS-responsive hydrogels	Preserve physiological ROS signaling	[128,129,130]
Nanozymes	Dynamic ROS buffering	[120,121]
Oxygen-generating matrices	Restore redox balance indirectly	[122,123]

Abbreviations: ROS—Reactive Oxygen Species.

**Table 9:** Extracellular Vesicle-Based Biomaterial Platforms in Regenerative Wound Healing.

EV Source	Bioactive Cargo	Biomaterial Carrier	Regenerative Effect	Major Translational Limitation	References
Bone marrow MSC-derived EVs	miRNAs, VEGF, anti-inflammatory proteins	Hydrogel scaffold	Enhanced angiogenesis and epithelialization	Cargo heterogeneity	[148,149]
Adipose-derived MSC EVs	Angiogenic cytokines, growth factors	Electrospun nanofibers	Accelerated wound closure	Isolation variability	[150,151]
Hypoxia-preconditioned EVs	HIF-1α-associated miRNAs	Injectable hydrogel	Improved ischemic tissue repair	Standardization challenges	[152]
Engineered exosomes	Gene-edited regenerative cargo	Smart hydrogel systems	Programmable regenerative signaling	Regulatory complexity	[153]
Umbilical cord MSC EVs	Anti-fibrotic mediators	Collagen-based scaffold	Reduced scar formation	Manufacturing scalability	[154,155]
Macrophage-derived EVs	Immunomodulatory cytokines	Conductive biomaterials	Immune microenvironment regulation	Limited clinical validation	[156,157]

Abbreviations: EV—Extracellular Vesicle, HIF-1α—Hypoxia-Inducible Factor 1-Alpha, miRNA—MicroRNA, MSC—Mesenchymal Stem Cell, VEGF—Vascular Endothelial Growth Factor.

**Table 10:** Representative Clinical Studies Investigating Advanced Wound Dressings and Regenerative Biomaterials.

Dressing/Platform	Study Type	Clinical Indication	Major Findings	Limitations	Translational Status	References
Silver nanoparticle dressings	Randomized clinical trials	Chronic wounds, postoperative wounds	Reduced bacterial burden and infection rates; accelerated healing in selected patients	Potential cytotoxicity and delayed epithelialization	Clinically available	[170,171,172]
Hydrogel-based dressings	Prospective clinical studies	Diabetic foot ulcers, surgical wounds	Improved moisture balance, angiogenesis, and granulation tissue formation	Variable mechanical stability and degradation kinetics	Partial clinical adoption	[173,174,175,176]
Growth factor-loaded biomaterials	Early-phase trials	Chronic diabetic wounds	Enhanced epithelialization and vascularization	Short growth factor half-life and high cost	Limited clinical implementation	[177,178,179]
Extracellular vesicle-loaded hydrogels	Pilot translational studies	Chronic non-healing wounds	Accelerated wound closure and reduced inflammatory signaling	Lack of standardized EV isolation protocols	Experimental	[180,181,182]
Conductive bioelectronic dressings	Feasibility studies	Chronic ulcers and diabetic wounds	Real-time wound monitoring and improved healing assessment	Device integration and calibration challenges	Early translational stage	[183,184,185]
Oxygen-generating biomaterials	Small cohort studies	Ischemic and diabetic wounds	Improved tissue oxygenation and angiogenesis	Limited long-term safety data	Experimental	[186,187,188]
Negative-pressure wound therapy plus topical oxygen therapy	Meta-analysis	Chronic and diabetic wounds	Improved healing outcomes compared with single-modality therapy	Variable treatment protocols	Emerging combination strategy	[189]
Antimicrobial peptide dressings	Phase I/II studies	Infected chronic wounds	Broad-spectrum antimicrobial activity with reduced resistance potential	Peptide instability and manufacturing cost	Early clinical development	[190,191,192]
Electrospun nanofibrous scaffolds	Translational pilot studies	Burns and reconstructive wounds	Enhanced cell migration and ECM remodeling	Scalability and sterilization challenges	Preclinical-to-translational stage	[193,194,195]
Smart sensor-enabled dressings	In vivo validation study	Remote wound monitoring	Continuous physiological monitoring with early detection of wound deterioration	Device integration and calibration challenges	Early translational stage	[196]

Abbreviations: ECM—extracellular matrix, EV—Extracellular Vesicle.

**Table 11:** Biomarker-guided selection of advanced wound dressing platforms.

Wound Phenotype	Candidate Biomarkers	Dominant Biological Defect	Preferred Dressing Strategy	Rationale	References
Highly inflamed wound	IL-1β, TNF-α, IL-6, NLRP3 activation	Persistent innate immune activation	Immunomodulatory hydrogel or cytokine-responsive scaffold	Promotes inflammatory resolution without systemic immunosuppression	[29,30,31,32,33,34,35,36,37,38,45,46,62,63]
Oxidative-stress-dominant wound	ROS, 8-OHdG, lipid peroxidation products, low GSH/GSSG ratio	Redox imbalance and cellular injury	ROS-responsive hydrogel or nanozyme dressing	Restores redox homeostasis while preserving physiological ROS signaling	[42,43,44,45,46,47,48,49,50,51,52,53,54,55,56,57,86,87,88,97,98,99,108,109]
Ischemic/hypoxic wound	Low TcPO_2_, HIF-1α, lactate accumulation	Oxygen deficit and impaired angiogenesis	Oxygen-generating matrix or pro-angiogenic scaffold	Supports ATP production, endothelial survival, and neovascularization	[49,50,51,52,53,54,55,56,57,72,73,74,75,91]
Diabetic wound	Hyperglycemia, AGEs, impaired macrophage transition, high MMPs	Metabolic inflammation and ECM degradation	Glucose-responsive matrix, MMP-responsive hydrogel, EV-loaded dressing	Targets diabetic microenvironment rather than only surface closure	[39,40,41,72,73,74,75,95,96,97,98,99,101,102]
Senescent wound	p16INK4a, p21, SASP cytokines, MMP overexpression	Fibroblast/endothelial senescence	Senolytic or senomorphic biomaterial	Reduces chronic inflammatory secretome and restores regenerative capacity	[112,113,114]
Biofilm-dominant wound [105,106,107]	Bacterial burden, quorum-sensing molecules, persistent exudate	Polymicrobial biofilm and immune evasion	Anti-biofilm nanoparticle dressing, bacteriophage-loaded scaffold	Disrupts biofilm architecture and improves antimicrobial susceptibility	[105,106,107,118]
Fibrotic/scar-prone surgical wound	TGF-β, collagen I/III imbalance, YAP/TAZ activation	Excessive mechanotransduction and matrix contraction	Mechanoresponsive hydrogel or viscoelastic scaffold	Modulates fibroblast activation and scar-forming mechanical memory	[2,4,58,59,60,61,66,67,110,111]
High-risk postoperative wound	Obesity, diabetes, immunosuppression, high SSI risk	Combined immune, metabolic, and microbial vulnerability	Multifunctional antimicrobial-immunomodulatory dressing	Provides early protection while supporting immune-regenerative repair	[5,6,7,8,9,10,11,12,13,14]

Abbreviations: 8-OHdG—8-Hydroxy-2′-deoxyguanosine; AGEs—Advanced Glycation End Products; ATP—Adenosine Triphosphate; ECM—Extracellular Matrix; EV—Extracellular Vesicle; GSH/GSSG—Reduced Glutathione/Oxidized Glutathione Ratio; HIF-1α—Hypoxia-Inducible Factor-1 Alpha; IL-1β—Interleukin-1 Beta; IL-6—Interleukin-6; MMPs—Matrix Metalloproteinases; NLRP3—NOD-, LRR- and Pyrin Domain-Containing Protein 3; ROS—Reactive Oxygen Species; SASP—Senescence-Associated Secretory Phenotype; SSI—Surgical Site Infection; TcPO_2_—Transcutaneous Partial Pressure of Oxygen; TGF-β—Transforming Growth Factor Beta; TNF-α—Tumor Necrosis Factor Alpha; YAP/TAZ—Yes-Associated Protein/Transcriptional Coactivator with PDZ-Binding Motif.

**Table 12:** Translational readiness and barriers to emerging wound-dressing platforms.

Platform	Evidence Maturity	Approximate Translational Readiness	Main Strength	Main Barrier	Priority Before Broad Adoption	References
Conventional advanced dressings	High clinical use	High	Practicality and availability	Limited biological activity	Better patient stratification	[15,16,17,18]
Hydrogel dressings	Moderate to high	High/moderate	Moisture control, drug delivery, ECM mimicry	Mechanical instability, variable degradation	Standardized formulations and comparative trials	[81,82,83,84,85,86,87,88,89,90,91,92,93,94,95,96,97,98,99]
Antimicrobial nanoparticle dressings	Moderate	Moderate	Local infection control	Cytotoxicity, resistance ecology, safety concerns	Long-term safety and microbiome-aware evaluation	[105,106,107,108,109]
Nanozyme-based dressings	Mostly preclinical	Low/moderate	Sustained catalytic redox regulation	Nanotoxicity and biodistribution	Biodistribution, degradation, and chronic safety studies	[108,109]
Oxygen-generating matrices	Preclinical to early clinical	Low/moderate	Addresses ischemia and ATP depletion	Controlled oxygen release	Human ischemic wound trials	[56,57,91]
EV-loaded biomaterials	Preclinical/early translational	Low/moderate	Acellular regenerative signaling	Cargo heterogeneity, potency assays, GMP production	Standardized EV isolation and potency criteria	[68,69,70,71,72,73,74,75]
Ferroptosis-targeted hydrogels	Early preclinical	Low	Mechanistically novel endothelial protection	Uncertain causal role in humans	Biomarker-driven validation	[59,100,101,102]
Conductive biomaterials	Preclinical/early translational	Low/moderate	Bioelectrical stimulation and cell migration	Device integration and reproducibility	Defined stimulation protocols	[79,80,89,90]
Smart bioelectronic dressings	Early feasibility	Low	Real-time monitoring and adaptive therapy	Cost, calibration, data reliability, regulation	Prospective clinical utility studies	[76,77,78,79,80]
AI-guided closed-loop systems	Conceptual/early prototype	Very low	Personalized adaptive wound care	Data quality, liability, validation	Multicenter datasets and regulatory frameworks	[76,77,78,79,80]

Abbreviations: AI, artificial intelligence; ATP, adenosine triphosphate; ECM, extracellular matrix; EV, extracellular vesicle; GMP, good manufacturing practice.

**Table 13:** Roadmap for the next-decade development of precision-regenerative wound dressings.

Development Priority	Scientific Rationale	Required Action	Expected Impact
Replace descriptive models with mechanistic phenotyping	Wounds differ biologically despite similar appearance	Use biomarkers, spatial profiling, and digital wound analytics	Better patient-dressing matching
Standardize potency assays	Advanced biologics vary between batches	Define release criteria for EVs, hydrogels, nanozymes, and biologics	Improved reproducibility
Improve clinical trial design	Small heterogeneous studies limit interpretation	Use stratified randomized trials with mechanistic endpoints	Stronger clinical evidence
Validate human-relevant models	Rodent contraction poorly predicts human repair	Use porcine models, human skin equivalents, organ-on-chip systems	Better translational predictability
Integrate cost-effectiveness early	Expensive dressings may fail despite efficacy	Include health-economic endpoints	Improved reimbursement probability
Develop regulatory pathways for combination products	Smart dressings combine devices, drugs, biologics, and software	Early regulatory consultation	Faster clinical translation
Build AI-ready wound datasets	Closed-loop systems require robust data	Multicenter imaging, biomarker, and outcome datasets	Reliable predictive wound management
Move from wound closure to functional restoration	Closure alone does not equal tissue quality	Measure scar quality, tensile strength, perfusion, recurrence, and quality of life	More meaningful clinical outcomes

Abbreviations: AI, artificial intelligence; EVs, extracellular vesicles.

## Data Availability

No new data were created or analyzed in this study. Data sharing is not applicable to this article.

## Abbreviations

The following abbreviations are used in this manuscript:

AI	Artificial Intelligence
Akt	Protein Kinase B
AMPK	AMP-Activated Protein Kinase
ATP	Adenosine Triphosphate
CRISPR	Clustered Regularly Interspaced Short Palindromic Repeats
ECM	Extracellular Matrix
EGF	Epidermal Growth Factor
EV	Extracellular Vesicle
FAK	Focal Adhesion Kinase
FGF	Fibroblast Growth Factor
GMP	Good Manufacturing Practice
GPX4	Glutathione Peroxidase 4
HIF-1α	Hypoxia-Inducible Factor 1 Alpha
IL-1β	Interleukin-1 Beta
IL-4	Interleukin-4
IL-6	Interleukin-6
MMP	Matrix Metalloproteinase
MMPs	Matrix Metalloproteinases
mTOR	Mammalian Target of Rapamycin
MSC	Mesenchymal Stem Cell
NETs	Neutrophil Extracellular Traps
NF-κB	Nuclear Factor Kappa B
NLRP3	NOD-, LRR-, and Pyrin Domain-Containing Protein 3
Nrf2	Nuclear Factor Erythroid 2–Related Factor 2
PDGF	Platelet-Derived Growth Factor
PEDOT	Poly(3,4-ethylenedioxythiophene)(styrenesulfonate)
PI3K	Phosphoinositide 3-Kinase
ROS	Reactive Oxygen Species
SASP	Senescence-Associated Secretory Phenotype
SSI	Surgical Site Infection
STAT6	Signal Transducer and Activator of Transcription 6
TGF-β	Transforming Growth Factor Beta
TIMPs	Tissue Inhibitors of Metalloproteinases
TNF-α	Tumor Necrosis Factor Alpha
VEGF	Vascular Endothelial Growth Factor
YAP/TAZ	Yes-Associated Protein/Transcriptional Coactivator with PDZ-Binding Motif
